# Phenolic Compounds from *Carissa spinarum* Are Characterized by Their Antioxidant, Anti-Inflammatory and Hepatoprotective Activities

**DOI:** 10.3390/antiox10050652

**Published:** 2021-04-23

**Authors:** Ye Liu, Yongli Zhang, Felix Wambua Muema, Festus Kimutai, Guilin Chen, Mingquan Guo

**Affiliations:** 1 CAS Key Laboratory of Plant Germplasm Enhancement and Specialty Agriculture, Wuhan Botanical Garden, Chinese Academy of Sciences, Wuhan 430074, China; liuye@wbgcas.cn (Y.L.); zhangyongli@wbgcas.cn (Y.Z.); fwambua83@mails.ucas.ac.cn (F.W.M.); festokim81@mails.ucas.ac.cn (F.K.); glchen@wbgcas.cn (G.C.); 2 Sino-Africa Joint Research Center, Chinese Academy of Sciences, Wuhan 430074, China; 3 Innovation Academy for Drug Discovery and Development, Chinese Academy of Sciences, Shanghai 201203, China; 4 University of Chinese Academy of Sciences, Beijing 100049, China

**Keywords:** *Carissa spinarum*, polyphenols, DPPH assay, FRAP assay, COX-2 inhibition, hepatic protection

## Abstract

*Carissa spinarum* has been traditionally used for the treatment of various diseases due to its different pharmacological activities. However, the active compounds responsible for its potentially specific activities have rarely been explored. To this end, the ethyl acetate (EA) fraction was screened out and selected for further phytochemical isolation because of its promising activities in preliminary 2,2-diphenyl-1-picrylhydrazyl (DPPH), ferric reducing antioxidant power (FRAP) and COX-2 inhibition assays. As a result, 10 compounds (**1**−**10**), including a new one (**5**), were isolated, with eight of these being identified as phenolic compounds, as expected. Compound **9** possessed an IC_50_ value of 16.5 ± 1.2 µM, which was lower than that of positive control (vitamin C, 25.5 ± 0.3 µM) in the DPPH assay, and compounds **2**, **6**, **7** and **9** showed better total antioxidant capacity than vitamin C in the FRAP assay. Meanwhile, compounds **1**−**6** and **9** also had IC_50_ values of less than 1.0 µM, which was even better than the positive control indomethacin in the COX-2 inhibition assay. In this context, compounds **2** and **9** were further evaluated to exhibit clear hepatoprotective activities by improving the L02 cell viability and reducing ROS production using a H_2_O_2_-induced L02 cell injury model. This study provides initial evidence revealing the most potent phenolic compounds from the root bark of *C. spinarum* responsible for its antioxidant, anti-inflammatory and hepatoprotective activities.

## 1. Introduction

*Carissa spinarum* Linn. (*C*. *spinarum*), widely distributed in the tropical regions of Africa, Asia, Oceania and Indian Ocean islands [1,2], is an evergreen shrub with spines belonging to the Apocynaceae family [3]. It is also a well-known medicinal plant in some African countries, known as “magic shrub” for its wide range of indications, each part of which is traditionally used to treat different human ailments, such as cancers, malaria, gastric ulcers, diabetes, chronic joint pains, infections, etc. [2,4,5,6]. Many researchers have investigated the pharmacological activities of *C. spinarum* based on its claimed ethnopharmacological uses, and constantly emerging studies have revealed its potential biological activities in relation to its anti-inflammation, anti-tumor, antioxidant, antimicrobial, wound-healing, and anti-leishmanial effects, which have provided evidence for its extensive traditional applications [1,2]. However, its phytochemicals, especially the active compounds responsible for these activities about remain elusive, since the biological activity and chemical composition content can be affected by many factors, such as the weather, environmental stresses and even aphids or other pests [7,8]. In this work, we chose plant material collected from Mount Kenya (Kenya), and focused primarily on its antioxidant, anti-inflammatory, and hepatoprotective activities.

Oxidative stress refers to the imbalance between the excess production of reactive oxygen species (ROS) or oxidants and the antioxidant response in cells or tissues [9,10]. Excessive accumulation of ROS causes oxidative damage to cells, tissues and organs, which has been reported to alert many biological functions and involve different pathological processes [11,12,13,14]. Oxidative stress has been proven to be associated with the development of several chronic liver diseases, such as nonalcoholic steatohepatitis (NASH), hepatocellular carcinoma (HCC), hepatitis C virus infection (HCV), liver fibrosis and cirrhosis [15]. Therefore, antioxidative therapy has been considered as a promising strategy for the management of these liver diseases, for example, vitamin E and vitamin C have been given as two adjuvant medicine therapies for NASH and HCV infection [16,17]. Based on this background, plant-derived antioxidants were extensively utilized in disease prevention [18]. Several lignans with antioxidant activity from the stems of *C. spinarum*, collected from Thailand and India have been evaluated [19,20]. Hegde et al. studied the antioxidant capability of the ethanolic extract of the roots of *C. spinarum* from India using the CCl_4_ and paracetamol-induced hepatic rat model, and implied the correlations between its hepatoprotective activity and free radical scavenging properties, and also provided clues for the antioxidant potential of *C. spinarum* roots [21].

On the other hand, the available evidence has shown that inflammation, an imperative physiological reaction, plays an important role in the occurrence and development of many diseases, and during the pathological process, the inflammatory response could be activated by oxidative stress [22,23,24]. COX-2, a potent enzyme induced by cytokines, mitogens or endotoxins, can initiate inflammation and promote the synthesis of prostaglandins, which exert pro-inflammatory effects. More recently, emerging research has shown that COX-2 expression is altered in many liver related diseases, such as NAFLD, liver fibrosis and hepatocellular carcinoma, which causes the progression of hepatocellular injury, including inflammation, autophagy and cell senescence [23,25]. These studies have proposed a possible adjunctive treatment strategy targeting COX-2 for liver diseases. Taking the inflammation related indications together with the biological activities of *C. spinarum*, the possible mechanism targeting COX-2 and responsible chemical compounds could be explored.

In this context, this study aims to screen out and identify the chemical constituents responsible for the antioxidant, anti-inflammatory and hepatoprotective activities of the root bark of *C. spinarum*. To this end, preliminary antioxidant and anti-inflammatory activity screenings were conducted for the four partition extracts with different polarities from *C. spinarum*. This was done via DPPH, FRAP methods and a COX-2 inhibition assay, and the fraction with the best activities in both assays was selected for phytochemical isolation and identification. Then, the antioxidant and COX-2 inhibition capacities of these isolated compounds were further evaluated with the same assays. Finally, the compounds with the most potent antioxidant and COX-2 inhibition activities were further selected and tested for hepatoprotective activities using the H_2_O_2_-induced L02 injury model and ROS quantitative analysis.

## 2. Materials and Methods

### 2.1. Plant Material

The root bark of *C. spinarum* was collected from Mount Kenya (Kenya) and a voucher specimen (No. 20190501) was deposited in the herbarium of the Key Laboratory of Plant Germplasm Enhancement and Specialty Agriculture of Wuhan Botanical Garden, Chinese Academy of Sciences, Wuhan, China.

### 2.2. Reagents and Instrumentation

Analytically pure reagents (petroleum ether, dichloromethane, ethyl acetate, methanol and *n*-buthanol) for the extraction and isolation were purchased from Sinopharm Chemical Reagent Co., Ltd. (Shanghai, China) and chromatographically pure reagents for HPLC and HR-ESI-MS (methanol, acetonitrile, formic acid) were purchased from TEDIA Company Inc. (Fairfield, CA, USA). 2,2-diphenyl-1-picrylhydrazyl (DPPH), 1,3,5-tri(2-pyridyl)-2,4,6-triazine (TPTZ) for antioxidative activity assays, and vitamin C as the positive control were bought from Sigma-Aldrich Co. (St. Louis, MO, USA). The COX-2 inhibitor screening kit (fluorescence probe method) was purchased from Beyotime (Shanghai, China) and indomethacin was used as a positive control. The gels of Sephadex LH-20 (Pharmacia Fine Chemical Co., Ltd., Uppsala, Sweden), ODS (YMC, Tokyo, Japan), and silica gel (Qingdao Marine Chemical Inc., Qingdao, China) were used for column chromatography and YMC-Pack ODS-A C_18_ (5 µm, 250 × 10 mm i.d.; YMC, Tokyo, Japan) columns were used for HPLC on an Agilent 1100 system. The ^1^D and ^2^D NMR data were carried out on a Bruker-Avance-III 600 MHz (Bruker, Karlsruhe, Germany) with TMS as an internal standard and samples were dissolved in CD_3_OD. Optical rotations were measured on a PerkinElmer 341 polarimeter (Waltham, MA, USA). The OD values were tested on a Tecan Infinite M200 PRO multi-functional microplate reader (Männedorf, Switzerland).

### 2.3. Extraction and Isolation

The air-dried root bark (3.2 kg) was smashed, soaked in methanol, and ultrasonically extracted three times (30 min for each time) at room temperature. Then, the combined extracts were filtered, and concentrated under reduced pressure to obtain the total crude extracts (CE, 360.7 g), which were subsequently suspended in water and partitioned with petroleum ether (PE), dichloromethane (DCM), ethyl acetate (EA) and *n*-buthanol (*n*-BuOH). These four parts with different polarities were subject to further antioxidant activity screening, and the EA part was selected for phytochemical isolation and evaluation.

The EA part (14.0 g) was initially chromatographed over an MPLC column (ODS C_18_, 5 µm) with a stepwise MeOH–H_2_O gradient (from 50:50 to 90:10, 10.0 mL/min) to furnish five fractions (Frs. 1–5) and Fr. 1 was subjected to another MPLC column (ODS C_18_, 5 µm), and eluted with MeOH–H_2_O (from 20:80 to 55:45, 10.0 mL/min) to obtain Frs. 1.1–1.4. Fr. 1.1 was further segmented by reverse-phase preparative HPLC (MeOH–H_2_O, from 15:85 to 65:35, 10.0 mL/min), and then purified by reverse-phase semi-preparative HPLC to obtain compound **6** (1.0 mg) from Fr. 1.1.2 (MeOH–H_2_O–HCOOH, 15:85:0.05, 2.0 mL/min); compounds **5** (9.2 mg), **7** (1.1 mg) and **9** (4.6 mg) from Fr. 1.1.3 (MeOH–H_2_O–HCOOH, 21:79:0.05, 2.0 mL/min); compound **10** (7.9 mg) from Fr. 1.1.4 (MeOH–H_2_O–HCOOH, 30:70:0.05, 2.0 mL/min), and compound **8** (3.3 mg) from Fr. 1.1.5 (MeOH–H_2_O–HCOOH, 30:70:0.05, 2.0 mL/min). Further purification of Fr. 1.2 was performed successively by preparative HPLC (MeOH–H_2_O, from 20:80 to 65:35, 10.0 mL/min) and semi-preparative HPLC (MeOH–H_2_O–HCOOH, from 20:80:0.05 to 30:70:0.05, 2.0 mL/min), to yield compounds **1** (1.2 mg), **2** (1.5 mg), **3** (2.3 mg) and **4** (1.5 mg). The brief isolation flow chart is shown in Figure S1.

### 2.4. In Vitro Antioxidant Activity Determination

#### 2.4.1. DPPH Radical Scavenging Activity Assay

The 2,2-diphenyl-1-picrylhydrazyl (DPPH) radical scavenging abilities of the *C. spinarum* extracts and isolated compounds were assessed by the previously reported method [26,27]. The reaction mixtures containing 190 µL DPPH solution (100 µM) and 10 µL samples (extracts or compounds) or positive control (vitamin C) solutions with various concentrations or blank control (methanol) were placed in the 96-well plate, and incubated in darkness at room temperature for 30 min. Then, the absorbance of the reaction mixture was measured by the microplate reader at 517 nm. All groups were tested in triplicate and the DPPH radical scavenging activity was calculated by the following equation:

DPPH-radical scavenging percentage (%) = [(A_control_ − A_sample_)/A_control_] × 100% (1)

A_control_ and A_sample_ represent the absorbance of the blank control and samples or positive control, respectively. Additionally, the IC_50_ values, which were defined as the effective sample concentration when DPPH radical was scavenged by 50%, were calculated by the GraphPad Prism software.

#### 2.4.2. Ferric Reducing Antioxidant Power (FRAP) Assay

The FRAP assay was performed according to the methodology reported in [26,27] with minor modifications. Briefly, the FRAP working solution, composed of 300 mM acetate buffer (pH 3.6), 10 mM TPTZ solution and 20 mM FeCl_3_·6H_2_O solution in a ratio of 10:1:1 *(v*/*v*/*v)*, was freshly prepared and warmed at 37 °C before use. Then, 190 µL FRAP working solution and 10 µL samples were added in 96-well plates and incubated at 37 °C for 10 min. The absorbance of the reaction mixture was collected by a microplate reader at 593 nm. A standard curve was established by FeSO_4_ at final concentrations ranging from 1.56 to 200 µM, and the total antioxidant activities of the extracts, compounds and positive controls (vitamin C) were calculated by the standard curve and expressed in terms of the FeSO_4_ value (µM). All the results were shown by the average values of the three biological replications.

### 2.5. COX-2 Inhibition Assay

COX-2 inhibition activity was screened using a commercial assay kit by the fluorescence probe method, following the manufacturer’s instructions, based on a two-step reaction: the substrate (arachidonic acid) was firstly catalyzed by COX-2 to generate PGG_2_, which was then further catalyzed by COX-2 to produce PGH_2_. During the second step, the probe without fluorescence added in the reaction system was simultaneously catalyzed to probe with strong fluorescence. Briefly, three groups were set for the assay, including a blank control, 100% enzyme activity control and sample group. 75 µL COX-2 assay buffer (80 µL for the blank control group), 5 µL COX-2 cofactor solution, 5 µL COX-2 work solution (rhCOX-2, except for the blank control group) and 5 µL sample or positive control (indomethacin) solution with various concentrations (equal amount of solvent for blank control and 100% enzyme activity control group) were added and mixed well in a 96-well black plate. After incubation for 10 min at 37 °C, 5 µL COX-2 substrate and 5 µL COX-2 probe were added and incubated in darkness for 10 min. Then, the fluorescence intensity was recorded by microplate reader with the excitation wavelength at 560 nm and the emission wavelength at 590 nm. The COX-2 activity inhibition percentage was calculated by the following equation:

COX-2 activity inhibition (%) = [(F_100% activity control_ − F_sample_)/(F_100% activity control_ − F_blank control_)] × 100% (2)

where F_100% activity control_, F_sample_ and F_blank control_ represent the fluorescence intensity of the 100% enzyme activity control, samples or positive control, and blank control, respectively. Additionally, the IC_50_ value, which was defined as the effective sample concentration when COX-2 activity was inhibited by 50%, was calculated by the GraphPad Prism software.

### 2.6. Hepatoprotective Property Assay

#### 2.6.1. Establishment of H_2_O_2_-Induced Oxidative Stress Model in L02 Cells

The L02 cells were purchased from BeNa Culture Collection Company (BNCC, Beijing, China) and cultured in RPMI-1640 medium supplemented with 10% fetal bovine serum (FBS) and 1% penicillin–streptomycin solution at 37 °C under 5% CO_2_ in a humidified incubator. The H_2_O_2_ induced oxidative stress model of L02 cell was established according to the reported method with slight modifications [28,29,30]. Firstly, 120 µL L02 cell suspension (1 × 10^4^ cells) was seeded in a 96-well plate and incubated for 24 h, followed by the addition of 15 µL PBS buffer (corresponding to the addition of tested samples). A total of 12 h later, 15 µL H_2_O_2_ solution with different concentrations (final concentration 100, 200, 250, 300, 350, 400, 450 µM) or an equal amount of PBS (normal control group) was added and treated for 2, 4 or 8 h to determine the best H_2_O_2_ concentration and stimulation time. The cell viability was evaluated by the SRB test kit (BestBio, Shanghai, China) following the manufacturer’s instructions.

#### 2.6.2. Hepatoprotective Activity of Select Compounds on L02 Cells against H_2_O_2_-Induced Oxidative Stress

According to the above result, after the 24 h incubation, the L02 cells were pretreated with 15 µL samples of various concentrations or PBS (H_2_O_2_-induced model and normal control group) for 12 h. Then, 15 µL H_2_O_2_ solution (300 µM) or PBS (normal control group) was added and maintained for 4 h. Finally, the cell viability was tested by the SRB method and expressed as a percentage of the normal control group. Each group was performed in triplicate.

#### 2.6.3. Quantitation and Photography of ROS in Hepatoprotective Assays

The quantitation of intracellular ROS was detected by the ROS assay kit purchased from Beyotime (Shanghai, China) following the manufacturer’s instructions. The cell pretreatment was the same as hepatoprotective activity screening. Four hours later, after the addition of H_2_O_2_, the cells were incubated with 10 µM DCFH-DA (final concentration in RPMI-1640 medium) for 30 min, and were then washed three times with medium. Finally, the fluorescence intensity was recorded by a microplate reader with the excitation wavelength at 488 nm and the emission wavelength at 525 nm and then the fluorescence photos were taken by a cell imaging multi-mode reader (Cytation 1, BioTek, Winooski, VT, USA). Each group was performed in triplicate and the fluorescence values were calibrated by the corresponding cell viabilities using the SRB method.

### 2.7. Statistical Analysis

All data in this work are expressed as means ± standard deviation (SD). The percentages of scavenging or inhibition rates were transformed to log values and IC_50_ values were calculated by GraphPad Prism 5 (GraphPad Software Inc., San Diego, CA, USA) using log (inhibitor) vs. normalized response-variable slope method. The standard curves used in FRAP assays were generated by Microsoft Excel (Microsoft Corporation, Redmond, WA, USA). The statistical analyses in this work were performed by SPSS 26.0 (SPSS Inc., Chicago, IL, USA) using one-way ANOVA Duncan’s multiple range test at the significance level of 0.05.

## 3. Results and Discussion

### 3.1. In Vitro Antioxidant Activity Assays of C. spinarum Extracts

The radical scavenging abilities of the crude extract (CE), PE, DCM, EA and *n*-BuOH fractions from the root barks of *C. spinarum* were evaluated by DPPH assays, which is a widely used method based on the stable organic nitrogen radical DPPH with a strong purple color in solutions. As shown in Table 1, after enrichment by extracting with different solvents with gradient polarity, the DCM, EA and *n*-BuOH fractions exhibited potential radical scavenging activity in a dose-dependent manner with the IC_50_ values of 136.0 ± 7.6, 31.8 ± 1.3 and 92.4 ± 8.6 µg/mL, respectively; whereas the crude extract and PE fraction exhibited very weak radical scavenging activities at the highest tested concentrations (21.7 ± 2.2% at 500 µg/mL for CE and 9.9 ± 0.8% at 500 µg/mL for PE).

To further evaluate the antioxidant activity of *C. spinarum*, the total antioxidant capacity of each extract was also evaluated by the ferric reducing antioxidant power (FRAP) assay, based on the mechanism of electron transfer reducing the ferric TPTZ to ferrous TPTZ. In this way, the total antioxidant potential of each sample was determined by using a ferrous ion (FeSO_4_) standard curve and expressed as the corresponding Fe^2+^ concentration, calculated by the regression equation. Similar to the results in the DPPH assays, the EA fraction exhibited the best antioxidant activity, closely followed by *n*-BuOH and DCM fractions, while the crude extract and PE fraction showed weak activities (Table 1).

According to the action mechanism of these two methods, the DPPH assay reflected the DPPH radical scavenging capacity, while the FRAP assay reflected the reduction ability via electron transfer, which was one of the radical neutralization pathways [31]. Generally, these two methods should be used together to assess the antioxidant activities. Taking both results into consideration, the crude extract and four extract fractions from *C. spinarum* with gradient polarity exhibited various antioxidant capacities with the same order, as follows: EA > *n*-BuOH > DCM > CE > PE. As shown in Table 1, the EA part was the most promising fraction and the FRAP value was better than vitamin C. In addition, the elimination of low polarity constituents improved the antioxidant capacity of samples. Similar screenings have shown that the fruit extract [32], the leaf ethanol extract and the stem chloroform extract of *C. spinarum* all possessed good antioxidant activities in the DPPH assay [20,33]. To the best of our knowledge, the antioxidant activity of root bark extract of *C. spinarum* was first evaluated in this work.

### 3.2. In Vitro COX-2 Inhibition Assays of C. spinarum Extracts

**Figure 1:**
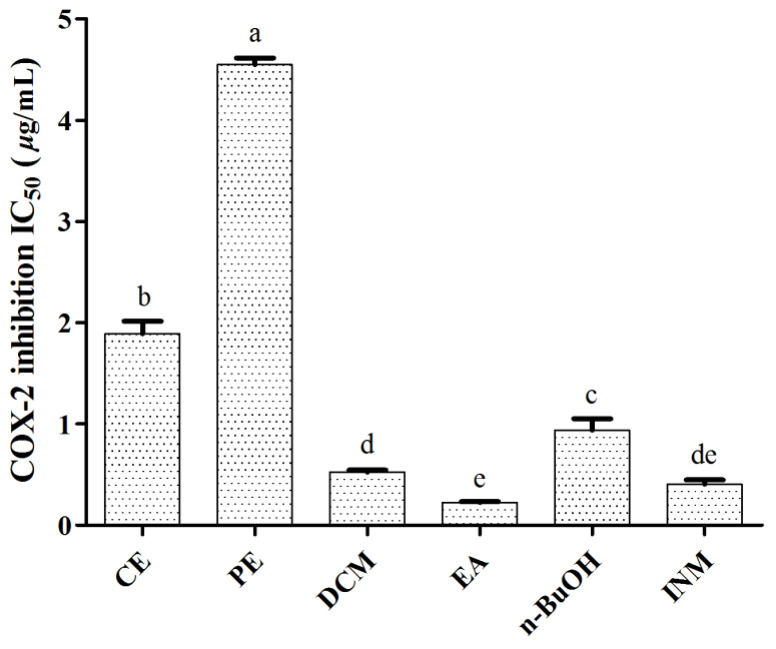
COX-2 inhibition activities of CE, PE, DCM, EA and *n*-BuOH fractions of *C. spinarum*. Indomethacin (INM) was used as a positive control. Data are expressed as means ± SD (*n* = 3). Means labeled by different letters (a–e) are significantly different at a level of *p* < 0.05 (*n* = 3) by one-way ANOVA DMRT.

*C. spinarum* has been traditionally used for inflammation, pain and fever related disorders. COX-2 is well known as the rate-limiting enzyme that catalyzes arachidonic acid to prostaglandins, which plays important roles in inflammation in many pathophysiologic processes [34]. Therefore, the COX-2 inhibition activity was firstly evaluated by the commercial COX-2 inhibitor screening kit in this work. As shown in Figure 1, the crude extract and four fractions showed COX-2 inhibition activities with varying degrees, among which, the DCM (0.5 ± 0.0 µg/mL) and EA (0.2 ± 0.0 µg/mL) fractions showed statistically equal COX-2 inhibitory activities to the positive control (INM, 0.4 ± 0.1 µg/mL). The EA fraction possessed the lowest IC_50_ value compared with INM and tested samples, followed by the DCM, *n*-BuOH, CE and PE fractions.

Despite the wide indication of *C. spinarum* in inflammation related diseases, relevant modern research is rare. The anti-inflammatory activity of the leaf extract of *C. spinarum* was speculated sue to its ability to attenuate formalin induced rat hind paw edema, but there was lack of direct evidence [35]. The significant COX-2 inhibition capacities of the DCM and EA fractions of *C. spinarum* tested in this work provided convincing evidence for its anti-inflammation applications. Meanwhile, the order of the COX-2 inhibition ability of these fractions was identical to those of the antioxidant results, which motivated us to explore the responsible ingredients.

### 3.3. Structure Elucidation of Isolated Compounds from C. spinarum

**Figure 2:**
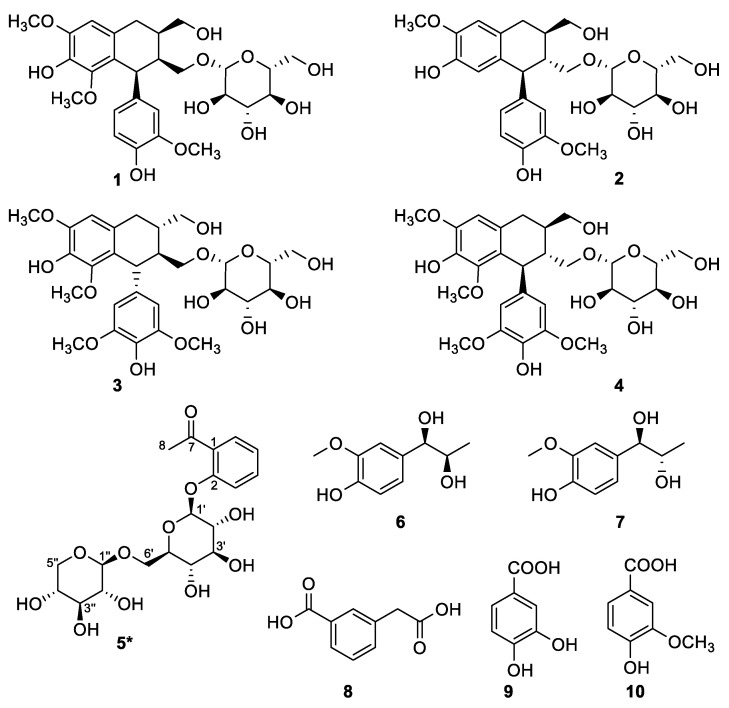
Chemical structures of compounds **1**–**10** isolated from *C. spinarum* (* new compound).

Guided by the antioxidant and COX-2 inhibition activity results above, the EA fraction was selected for further phytochemical isolation and identification, aiming to reveal the most active compounds responsible for both activities of interest. The detailed phytochemical investigation of the ethyl acetate (EA) fraction of the methanolic extract obtained from *C. spinarum* root bark led to the isolation of 10 compounds (**1**–**10**), including one undescribed one (**5**), as shown in Figure 2. Based on the comprehensive spectral analysis, their structures were elucidated as follows.

**Figure 3:**
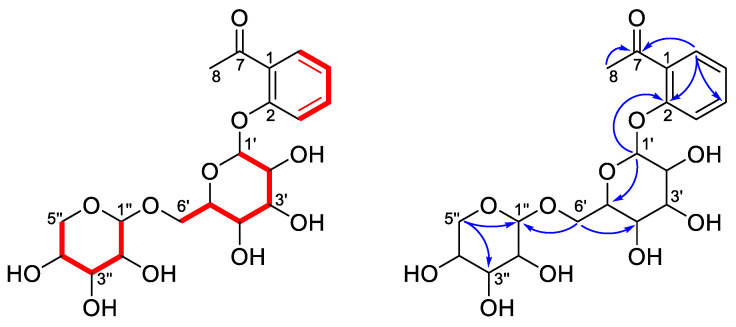
Key ^1^H–^1^H COSY (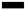) and HMBC (→) correlations of compound **5**.

Compound **5**, [α${]}_{D}^{20}$—51.6 (*c* 0.31, CH_3_OH), was obtained as a colorless oil and its molecular formula was determined to be C_19_H_26_O_11_ on the basis of the HR-ESI-MS analysis ([M + HCOO^−^] at *m*/*z* 475.1460, calcd. for 475.1452). The ^1^H NMR data of **1** (Table 2) displayed an *ortho*-disubstituted aromatic ring system at *δ*_H_ 7.67 (1H, dd, *J* = 7.5, 1.8 Hz, H-6), 7.56 (1H, ddd, *J* = 8.7, 7.5, 1.8 Hz, H-4), 7.39 (1H, dd, *J* = 8.7, 1.0 Hz, H-3) and 7.11 (1H, td, *J* = 7.5, 1.0 Hz, H-5), supported by the corresponding ^13^C NMR data (Table 2) and ^1^H–^1^H COSY correlations of H-3/H-4/H-5/H-6 (Figure 3) [36]. Additionally, the singlet methyl group at *δ*_H_ 2.69 (3H, s, H-8), along with the carbonyl carbon signal at *δ*_C_ 202.5, was disclosed to be an acetyl group attached to C-1 based on the HMBC correlations of H-6, H-8/C-7 (Figure 3). The remaining characteristic ^1^H NMR signals from *δ*_H_ 3.13 to 5.04, combined with the oxygenated carbons, contributed to two sugar units. The chemical shifts of these oxygenated carbons from *δ*_C_ 66.9 to 105.4 were the same as the ones reported for the disaccharide chain consisting of glucose and xylose [37,38], which was further verified by the ^1^H–^1^H COSY and HMBC correlations shown in Figure 3, by which the assignments of the sugar moieties are well defined in Table 1. The inference of interglycosidic linkage involving C-6′ and C-1″ between glucose and xylose was reasoned by the HMBC long-range correlations from H-6′a and H-6′b to C-1″. Likewise, the attachment of the sugar chain at C-2 was established by the HMBC cross-peak between H-1′ and C-2. The large coupling constants of *J*_1′, 2′_ (7.7 Hz) and *J*_1″,2″_ (7.5 Hz), along with the NOESY correlations of H-1′/H-3′ (Figure S9) and H-1″/H-3″, suggested the *β*-configuration of the anomeric centers for both sugar units [36,37,38]. Hence, compound **5** was determined to be acetophenone-2-*O*-*β*-xylopyranosyl-(1→6)-*O*-*β*-glucopyranoside.

These known compounds were identified as (6*R*,7*S*,8*S*)-7a-[(*β*-D-glucopyranosyl)-oxy]-1-methoxyisolariciresinol (**1**) [39], (+)-isolariciresinol 3a-*O*-*β*-D-glucopyranoside (**2**) [40], (-)-lyoniresinol 3*α*-*O*-*β*-D-glucopyranoside (**3**) [41], (+)-lyoniresinol 3*α*-*O*-*β*-D-glucopyranoside (**4**) [41,42], erythro-1-(3-methoxy-4-hydroxy-phenyl)-propan-1,2-diol (**6**) [43], threo-1-(3-methoxy-4-hydroxy-phenyl)-propan-1,2-diol (**7**) [43,44], 3-carboxymethyl-benzoic acid (**8**) [45], protocatechuic acid (**9**) [46], vanillic acid (**10**) [46], by comparison of their spectral data with those reported in the literatures.

### 3.4. In Vitro Antioxidant Activity Assays of Isolated Compounds

**Figure 4:**
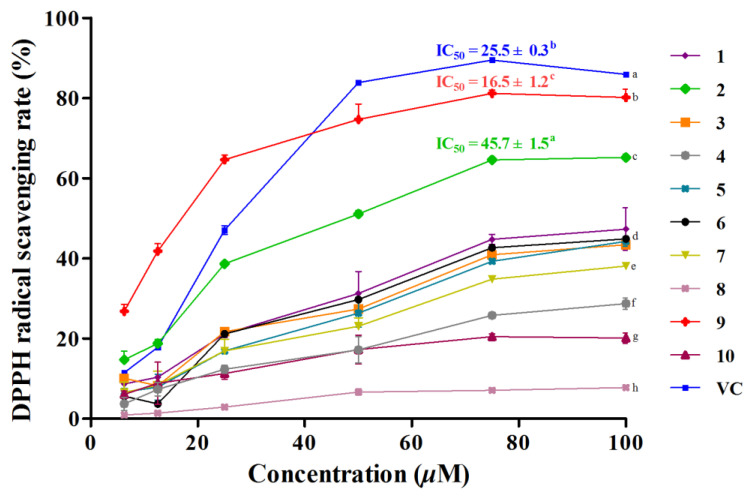
Radical scavenging activities of compounds **1**−**10** by DPPH assays. Vitamin C (VC) was used as positive control. Data are expressed as means ± SD (*n* = 3). IC_50_ values labeled by different letters (a–c) and the DPPH radical scavenging rate at 100 µM labeled by different letters (a–h) were significantly different at a level of *p* < 0.05 (*n* = 3) by one-way ANOVA DMRT.

**Figure 5:**
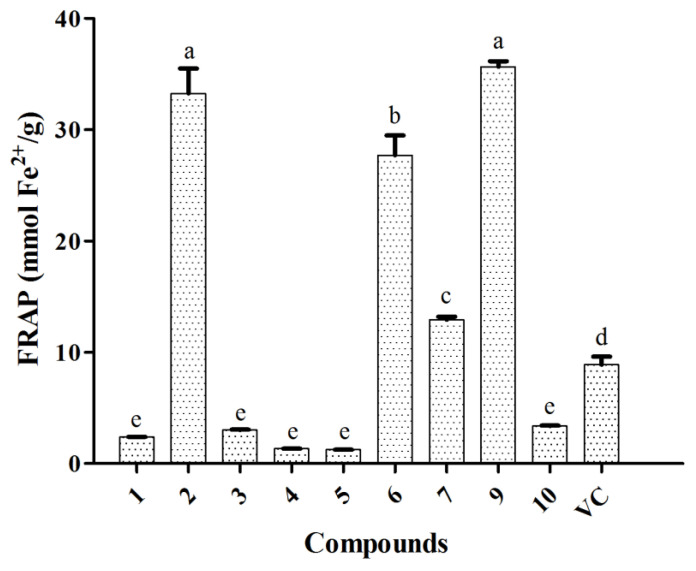
Total antioxidant activities of compounds **1**−**7** and **9**−**10** by FRAP assays. VC was used as positive control. Compound **8** did not show any activities in FRAP assay. Data are expressed as means ± SD (*n* = 3). Means labeled by different letters (a–e) were significantly different at a level of *p* < 0.05 (*n* = 3) by One-Way ANOVA DMRT.

All the isolated compounds were tested for their antioxidant activities by DPPH and FRAP methods, as mentioned above, with final concentrations ranging from 6.25 to 100 µM. In the DPPH experiment, compounds **1**−**7** and **9**−**10** showed radical scavenging activities with various degrees, whereas **8** did not exhibit any potential, with a scavenging rate lower than 10% at the highest tested concentration. As demonstrated in Figure 4, compounds **2** and **9** exhibited stronger radical scavenging capacities than other compounds, among which, **9** was even better than the positive control (vitamin C, IC_50_ value of 25.5 ± 0.3 µM) with IC_50_ values of 16.5 ± 1.2 µM, while in the high concentration groups, no compound possessed better DPPH radical scavenging rate than the positive control. In the FRAP experiment, all the isolates displayed similar results to the DPPH assay with dose-dependent reducing capacities at all concentration groups. As shown in Figure 5, compounds **1**−**7** and **9**−**10** showed total antioxidant activities with varying levels, ranking as follows: **9** > **2** > **6** > **7** > Vc > **10** > **3** > **1** > **4** > **5**, among which, compounds **2**, **6**, **7** and **9** possessed higher reducing capacities than the positive control, vitamin C. As known, the antioxidant capacities of phenolic compounds were determined by the number and arrangement of the phenolic hydroxyl groups in their structures [47]. Accordingly, in our work, two non-phenolic compounds (**5** and **8**) showed no obvious antioxidant potential, while compounds **2** and **9**, possessing two phenolic hydroxyl groups, exhibited the best antioxidant activities in both methods. With regard to compounds **1**, **3** and **4**, the poor antioxidant potential might be attributable to the substituent position of methoxy groups, which affected the donation of their active hydrogen.

### 3.5. COX-2 Inhibition Activity Assays of Isolated Compounds

**Figure 6:**
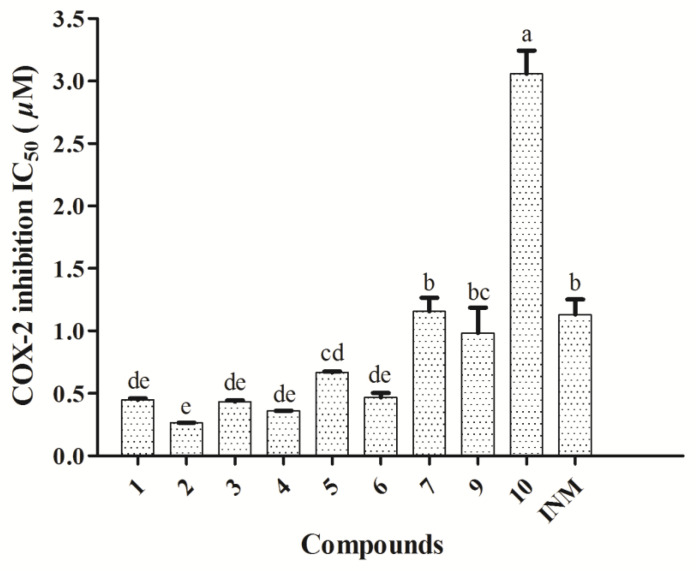
IC_50_ values of compounds **1**−**7** and **9**−**10** in the COX-2 activity inhibition assay. INM was used as the positive control. Compound **8** did not show any activities in this assay. Data are expressed as means ± SD (*n* = 3). Means labeled by different letters (a–e) were significantly different at a level of *p* < 0.05 (*n* = 3) by one-way ANOVA DMRT.

The anti-inflammatory activities of all the compounds was also evaluated by the COX-2 inhibitor screening method, with final concentrations ranging from 0.04 to 25 µM. As displayed in Figure 6, compounds **1**−**7** and **9**−**10** showed COX-2 inhibition capacities of varying degrees, ranking as follows: **2** **> 4 > 3 > 1 > 6 > 5 > 9 > INM > 7 > 10**, except for compound **8** with no COX-2 ability in all tested concentrations. Among these compounds, **1**−**6** and **9** exhibited promising activities with IC_50_ values under 1.0 µM, in particular, compound **2** with an IC_50_ value of 0.3 ± 0.0 µM, possessed a much better activity compared with indomethacin (IC_50_ = 1.1 ± 0.2 µM), which is the frequently used COX-2 inhibitor in the clinic. Among these compounds, all four lignan glycosides **1**−**4** showed significant COX-2 inhibition activities, which would explain the wide pharmacological benefits of lignans on inflammation related diseases. More interestingly, compounds **6** and **7** shared the same planar structure, but their COX-2 inhibition activities were statistically distinguished due to the different stereo-structures.

### 3.6. Hepatoprotective Properties of Select Compounds

#### 3.6.1. Establishment of H_2_O_2_-induced L02 Injury Model

**Figure 7:**
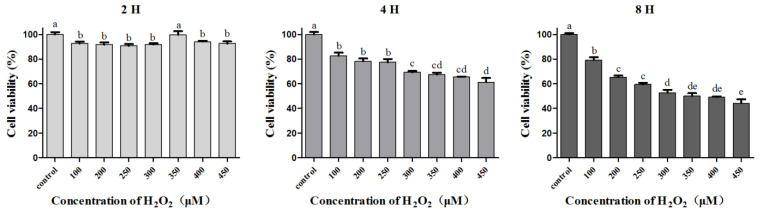
Effects of gradient concentrations and different treated times of H_2_O_2_ on L02 cells. Data are expressed as means ± SD (*n* = 3), and means labeled by different letters (a–e) were found to be significantly different at a level of *p* < 0.05 (*n* = 3) by one-way ANOVA DMRT.

H_2_O_2_ is one of the ROS molecules, which could induce oxidative stress, so the L02 cells were treated with a gradient of H_2_O_2_ concentrations for different times to establish the L02 oxidative stress injury model [28,30], and the results revealed that cell viability demonstrated little obvious change in the 2 h group at all tested H_2_O_2_ concentrations. Meanwhile, cell viability was significantly reduced to 61.3–82.7% and 44.2–79.0% in a H_2_O_2_ concentration-dependent manner in the 4 h and 8 h groups, respectively (Figure 7). In order to ensure a suitable cell survival rate and avoid irreversible cell injury or death, 300 µM of H_2_O_2_, treated for 4 h, was chosen for the following experiments [30].

#### 3.6.2. Protective Effects of Select Compounds on H_2_O_2_-Induced Injury in L02 Cells

**Figure 8:**
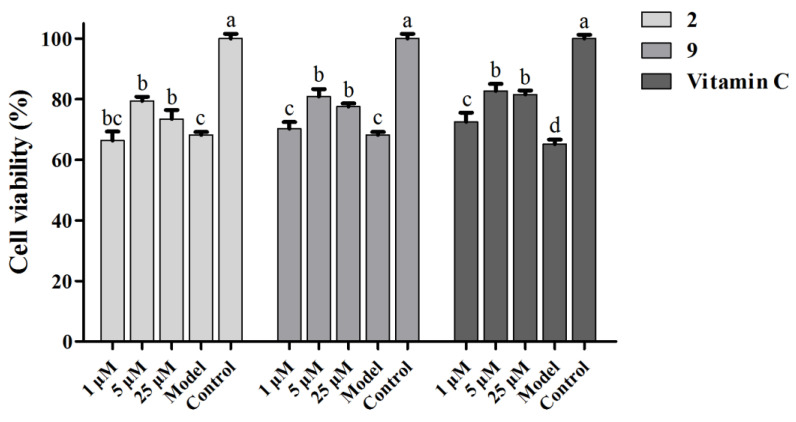
Protective effects of compounds **2** and **9** on H_2_O_2_-induced injury in L02 cells. Vitamin C was used as positive control. Data are expressed as means ± SD (*n* = 3), and means labeled with different letters (a–c) are significantly different at a level of *p* < 0.05 (*n* = 3) by one-way ANOVA DMRT.

Protective effects of selected compounds with significant antioxidant activities on hepatocytes were evaluated by L02 cells with H_2_O_2_-induced oxidative injury at concentrations ranging from 1 to 25 µM. As shown in Figure 8, the two tested compounds displayed statistically significant hepatoprotective effects against H_2_O_2_-induced L02 cell death, especially at 5 µM groups. The viabilities of L02 cells were improved from 68.2% in the H_2_O_2_ model group to 79.4% and 80.9% by compounds **2** and **9** at a concentration of 5 µM, respectively, whereas the positive control vitamin C from 65.2% to 82.7% at 5 µM. These results demonstrate that compounds **2** and **9**—with remarkable antioxidant and anti-inflammatory activities—also had a certain effect on the improvement of cell survival in the H_2_O_2_-induced L02 cell oxidative injury model, and interestingly, the compounds and positive control showed a parabolic-shape dose–response relationship within the tested concentration range.

**Figure 9:**
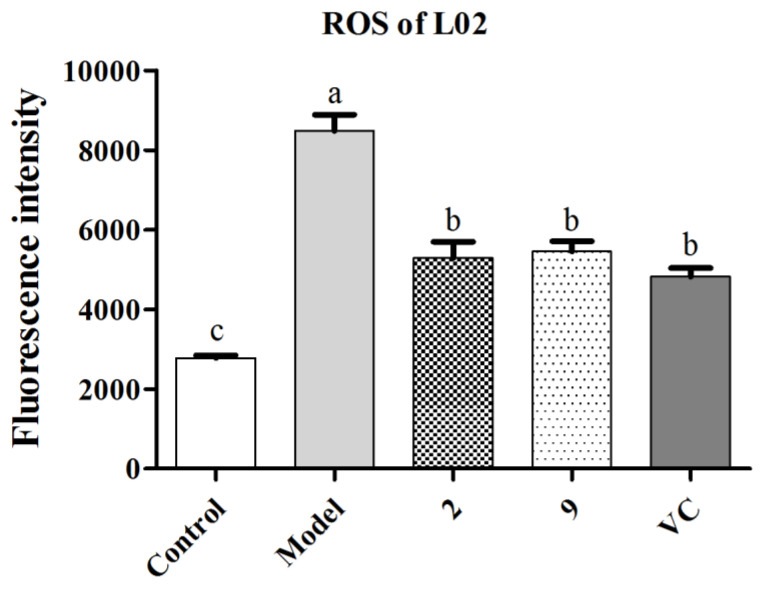
ROS production quantitation of compounds **2** and **9** at 5 µM on H_2_O_2_-induced injury in L02 cells. Vitamin C was used as positive control (5 µM). Data are expressed as means ± SD (*n* = 3) and means labeled by different letters (a–c) are significantly different at a level of *p* < 0.05 (*n* = 3) by one-way ANOVA DMRT.

**Figure 10:**
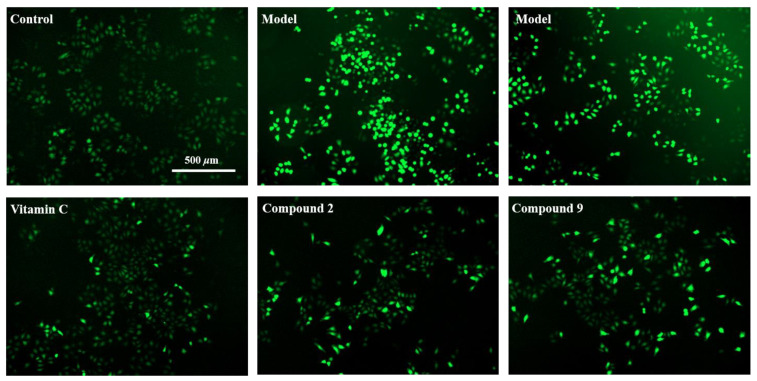
ROS production of compounds **2** and **9** at 5 µM on H_2_O_2_-induced injury in L02 cells showed in fluorescence images. Vitamin C was used as positive control (5 µM). Photographed by cell imaging multi-mode reader (Cytation 1, BioTek) at 4×, scale bar: 500 µm.

Next, the ROS level was detected based on the fluorescence probe DCFH-DA, which was finally oxidized by intracellular ROS to fluorescent DCF [28]. The relevant commercial assay kit was utilized as an indicator of the intracellular ROS level. Accordingly, as shown in Figure 9 and Figure 10, the ROS level of the H_2_O_2_-induced model group increased remarkably compared with the control group. Meanwhile, the groups treated with compounds **2**, **9** or the positive control at 5 µM (final concentration) exhibited statistically significant decreases in ROS quantity, which indicated that these two compounds could protect L02 cells from H_2_O_2_-induced injury by reducing ROS production.

Oxidative stress induced by excessive ROS production led to the dysfunction of hepatic homeostasis, which triggered liver injury, manifesting as an inflammation response, lipid peroxidation, DNA damage, and cell apoptosis, resulting in several chronic liver diseases [15,23]. As the results demonstrate, L02 cells treated with H_2_O_2_, causing a significant rise in ROS levels, suffered from cell death and morphological changes. Therefore, antioxidants are a potential class of compounds to treat liver diseases related to oxidative stress [16]. Compound **2** belongs to lignan with an aryltetralin structure type, which is a large group of antioxidative polyphenolic compounds. Lignans have comprised an important source of hepatoprotective agents, for example, bifendate, a widely used liver-protecting drug with less toxicity and fewer side effects in the clinic is derived from natural lignans. In return, numerous emerging lignans have been reported to show benefits to the liver in terms of reducing hepatotoxicity, hepatic steatosis, liver oxidative stress and inflammation [48,49]. Compound **2** was first isolated from *C. spinarum*, and its COX-2 inhibition ability and hepatic protection activity against H_2_O_2_-induced cell injury were also first disclosed in this work. As for compound **9**, a very common phenolic compound, known as protocatechuic acid (PCA), has been shown to demonstrate anti-inflammatory and hepatoprotective activity in rats or mice models [50], and our work supplemented the COX-2 inhibition and hepatic cell protection evidence in vitro.

## 4. Conclusions

Natural antioxidants and anti-inflammatory agents are attracting ever increasing attention. Potential compounds from traditional medicinal plants are now actively being sought throughout the world for their health benefits, especially in relation to the liver. *C. spinarum*, as a medicinal plant, has been empirically studied for its potential use in the treatment of liver-relevant diseases in Africa. In order to explore its active compounds that are responsible for its hepatoprotective activities, the antioxidant and anti-inflammatory activities of the extracts of *C. spinarum* root bark from Kenya were firstly evaluated to screen out the most promising fraction EA, and two phenolic compounds (**2** and **9**) were demonstrated to simultaneously possess good antioxidant and anti-inflammatory activities. More strikingly, these two compounds also demonstrated good hepatoprotective potential. Collectively, this current study has provided updated evidence for the traditional use of *C. spinarum*, and further explored two potent candidate compounds which could be responsible for the anti-inflammatory and hepatoprotective effects. More importantly, the potent fraction or compounds revealed in this work could be further developed as natural remedies for hepatocyte damage related applications in the near future.

## Figures and Tables

**Table 1:** Antioxidant activities of different extracts of *C. spinarum* tested by DPPH and FRAP assays.

Sample	DPPH ^1^	FRAP ^1^
	IC_50_ (µg/mL)	mmol Fe^2+^/g
CE	>500	0.2 ± 0.0 ^d^
PE	>500	0.2 ± 0.0 ^d^
DCM	136.0 ± 7.6 ^a^	3.0 ± 0.7 ^c^
EA	31.8 ± 1.3 ^c^	14.9 ± 2.4 ^a^
*n*-BuOH	92.4 ± 8.6 ^b^	4.1 ± 0.0 ^c^
Vitamin C	4.5 ± 0.1 ^d^	8.9 ± 1.2 ^b^

^1^ Data were expressed as means ± SD (*n* = 3). ^a–d^ Means labeled by different letters are significantly different at a level of *p* < 0.05 (*n* = 3) by one-way ANOVA Duncan’s multiple range test (DMRT).

**Table 2:** ^1^H NMR (600 MHz) and ^13^C NMR (125 MHz) data of compound **5** in CD_3_OD.

Position	*δ*_H_, (*J* in Hz)	*δ*_C_	Position	*δ*_H_, (*J* in Hz)	*δ*_C_
1		130.4	4′	3.41, dd (9.8, 8.9)	71.3
2		158.0	5′	3.70, ddd (9.8, 6.4, 2.0)	77.6
3	7.39, dd (8.7, 1.0)	117.6	6′a	4.13, dd (11.7, 2.0)	69.8
4	7.56, ddd (8.7, 7.5, 1.8)	135.3	6′b	3.79, dd (11.7, 6.4)	
5	7.11, td (7.5, 1.0)	123.4	1″	4.32, d (7.5)	105.4
6	7.67, dd (7.5, 1.8)	130.8	2″	3.21, dd (8.9, 7.5)	75.0
7		202.5	3″	3.28, t (8.9)	77.7
8	2.69, s	32.2	4″	3.48, m, overlap	71.2
1′	5.04, d (7.7)	102.4	5″a	3.84, dd (11.5, 5.4)	66.9
2′	3.55, dd (9.2, 7.7)	74.9	5″b	3.13, dd (11.5, 10.2)	
3′	3.48, m, overlap	78.1			

## Data Availability

Not applicable.

## Supplementary Materials

The following are available online at https://www.mdpi.com/article/10.3390/antiox10050652/s1, Figure S1: The isolation flow chart of compounds **1**–**10**, Figure S2: (+)-HR-ESI-MS spectrum of **5**, Figure S3: ^1^H NMR (600 MHz, CD_3_OD) spectrum of **5**, Figure S4: ^13^C NMR (125 MHz, CD_3_OD) spectrum of **5**, Figure S5: DEPT 135 (125 MHz, CD_3_OD) spectrum of **5**, Figure S6: HSQC spectrum of **5** in CD_3_OD, Figure S7: ^1^H-^1^H COSY spectrum of **5** in CD_3_OD, Figure S8: HMBC spectrum of **5** in CD_3_OD, Figure S9: NOESY spectrum of **5** in CD_3_OD.
