# Supplementary material for: Phenolic Compounds from Carissa spinarum Are Characterized by Their Antioxidant, Anti-Inflammatory and Hepatoprotective Activities
Source: Antioxidants (Basel). 2021 Apr 23;10(5):652. doi: 10.3390/antiox10050652 (PMC8145564; doi:10.3390/antiox10050652)

Supplementary Materials for

Phenolic Compounds Screened and Revealed out from Carissa spinarum with Potent Antioxidant, Anti-inflammatory and Hepatoprotective Activities

Ye Liu[1,2,3], Yongli Zhang[1,2,3], Felix Wambua Muema[1,2,3,4], Festus Kimutai[1,2,3,4], Guilin Chen[1,2,3], and Mingquan Guo[1,2,3,*]

[1] CAS Key Laboratory of Plant Germplasm Enhancement and Specialty Agriculture, Wuhan Botanical Garden, Chinese Academy of Sciences, Wuhan 430074, China; liuye@wbgcas.cn (Y.L.); zhangyl@wbgcas.cn (Y.Z.); fwambua83@gmail.com (F.W.M.); festokim81@gmail.com (F.K.); glchen@wbgcas.cn (G.C.)

[2] Sino-Africa Joint Research Center, Chinese Academy of Sciences, Wuhan 430074, China

[3] Innovation Academy for Drug Discovery and Development, Chinese Academy of Sciences, Shanghai 201203, China

[4] Graduate University of Chinese Academy of Sciences, Beijing 100049, China

* Correspondence: guomq@wbgcas.cn; Tel.: +86-027-87700850

Content

Figure S1. The isolation flow chart of compounds **1–10**

Figure S2. (+)-HR-ESI-MS spectrum of **5**

Figure S3. ^{1}H NMR (600 MHz, CD_3OD) spectrum of **5**

Figure S4. ^{13}C NMR (125 MHz, CD_3OD) spectrum of **5**

Figure S5. DEPT 135 (125 MHz, CD_3OD) spectrum of **5**

Figure S6. HSQC spectrum of **5** in CD_3OD

Figure S7. ^{1}H-^{1}H COSY spectrum of **5** in CD_3OD

Figure S8. HMBC spectrum of **5** in CD_3OD

Figure S9. NOESY spectrum of **5** in CD_3OD

Figure S1. The isolation flow chart of compounds **1–10**

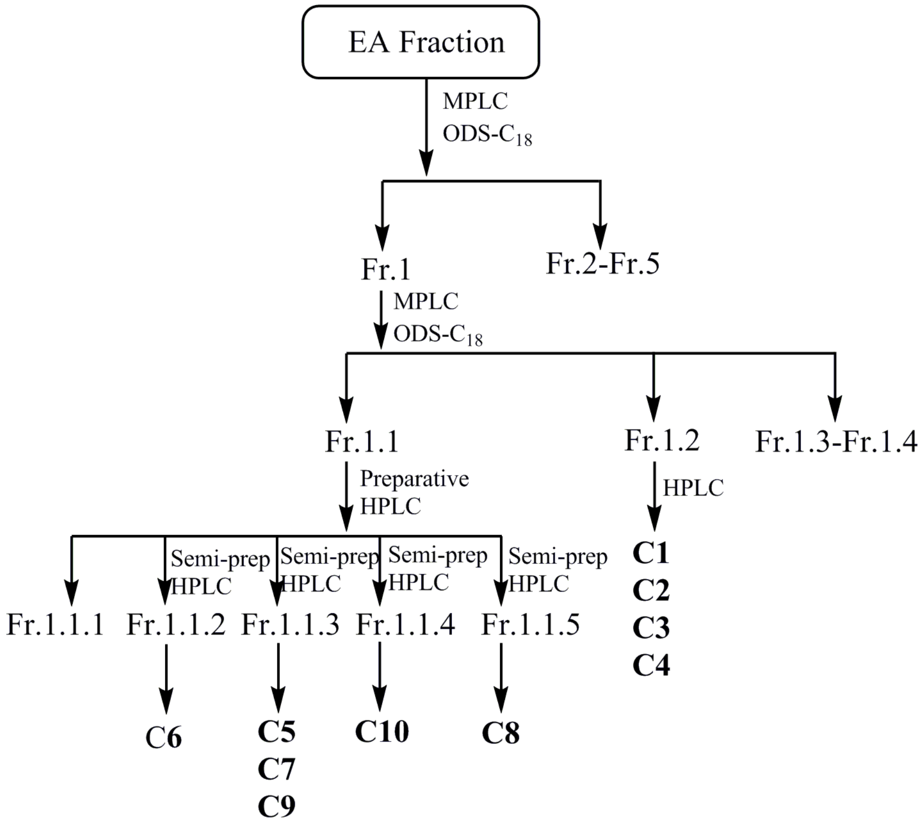

Figure S2. (+)-HR-ESI-MS spectrum of **5**

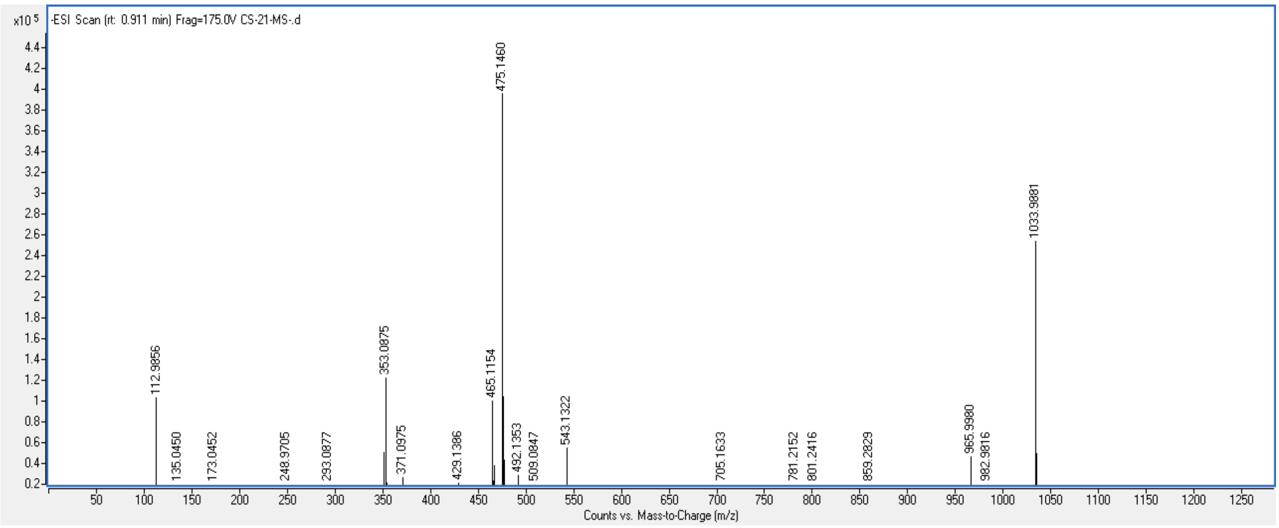

Figure S3. ^{1}H NMR (600 MHz, CD_3OD) spectrum of **5**

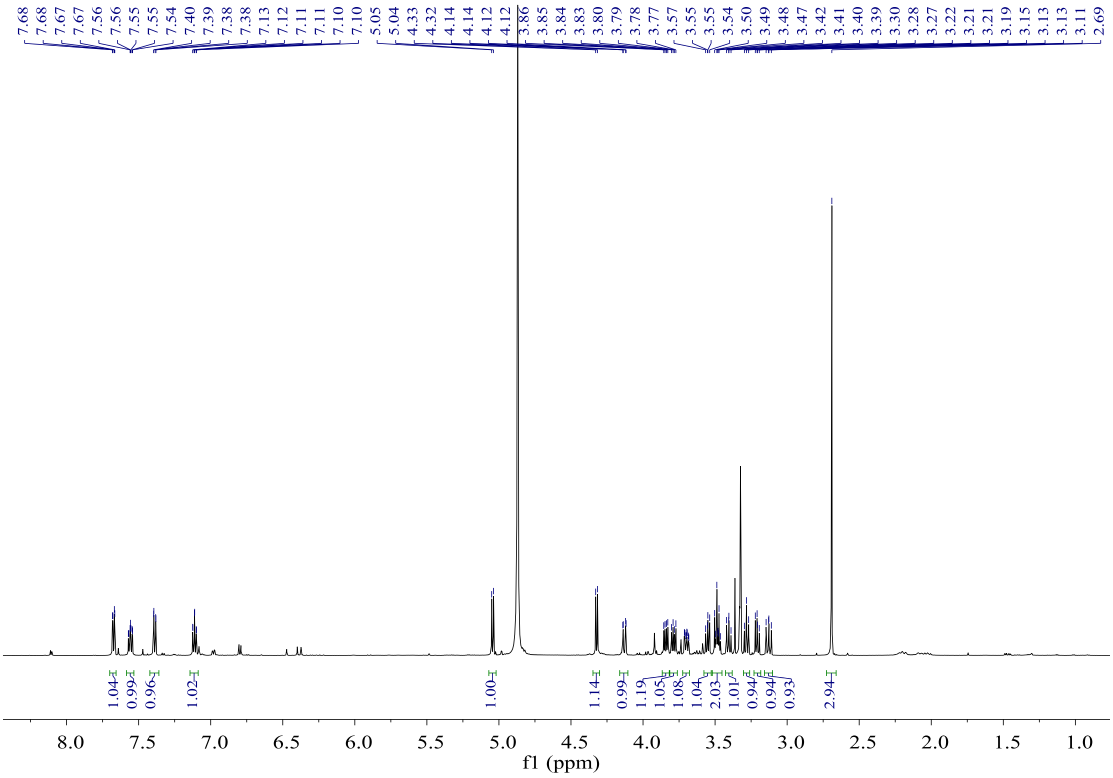

Figure S4. ^{13}C NMR (125 MHz, CD_3OD) spectrum of **5**

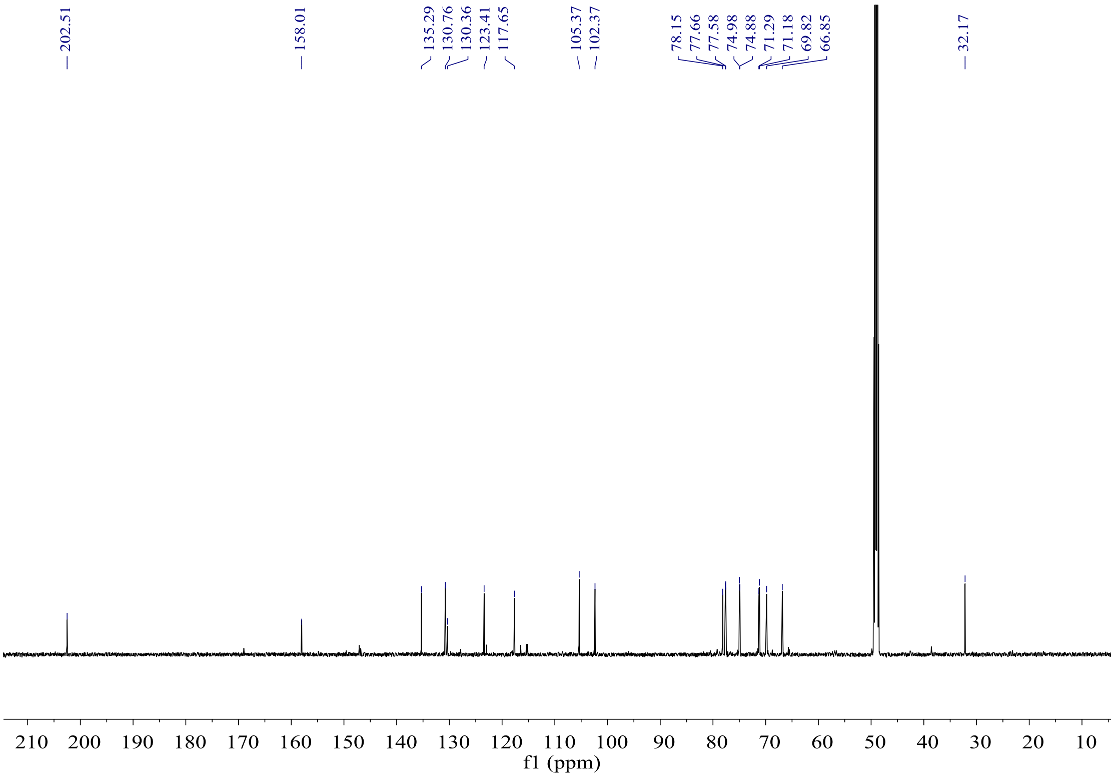

Figure S5. DEPT 135 (125 MHz, CD_3OD) spectrum of **5**

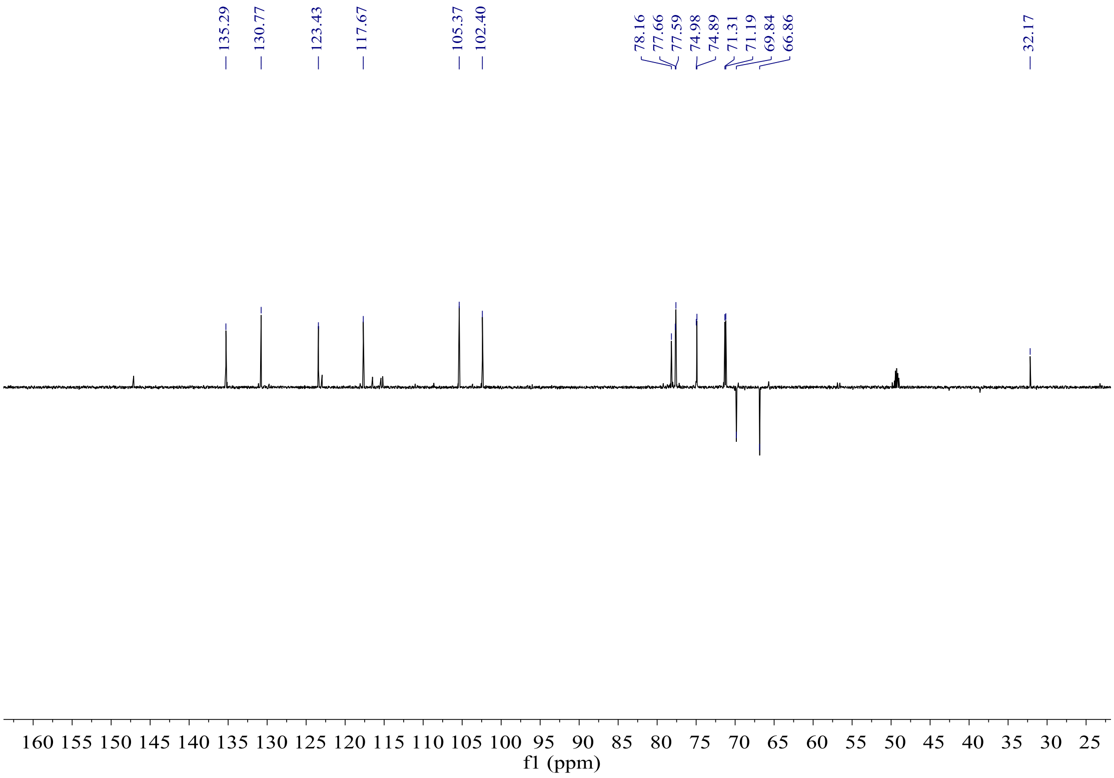

Figure S6. HSQC spectrum of **5** in CD_3OD

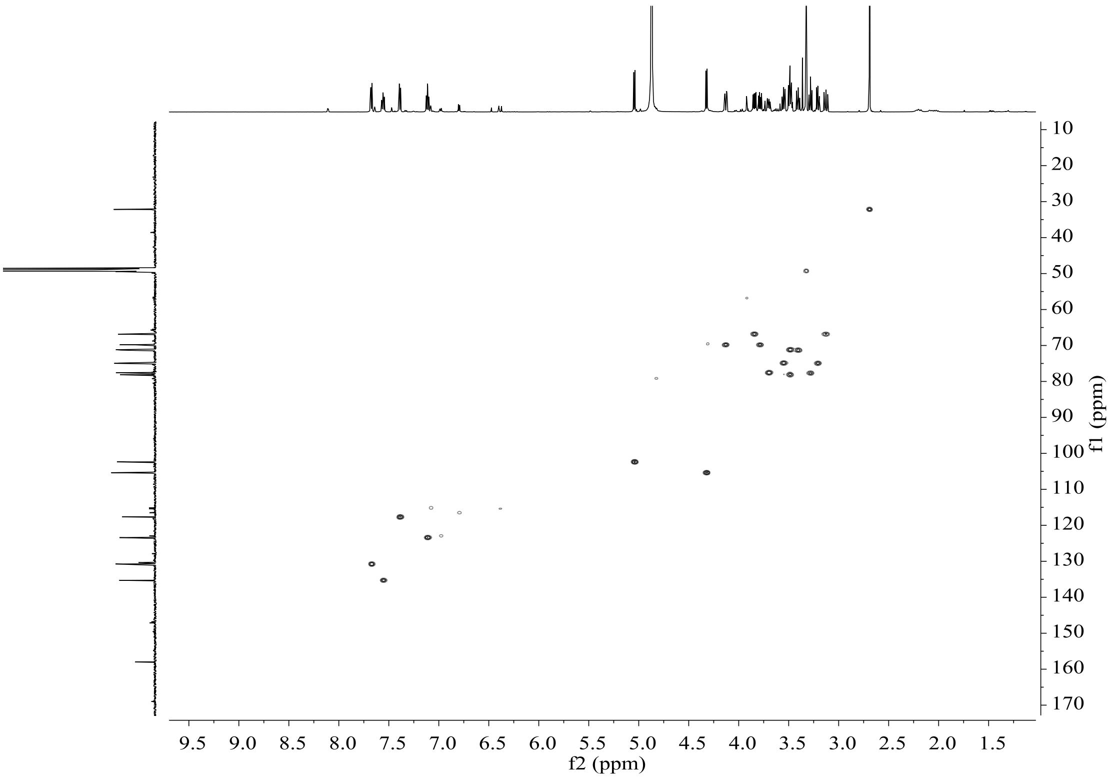

Figure S7. ^{1}H-^{1}H COSY spectrum of **5** in CD_3OD

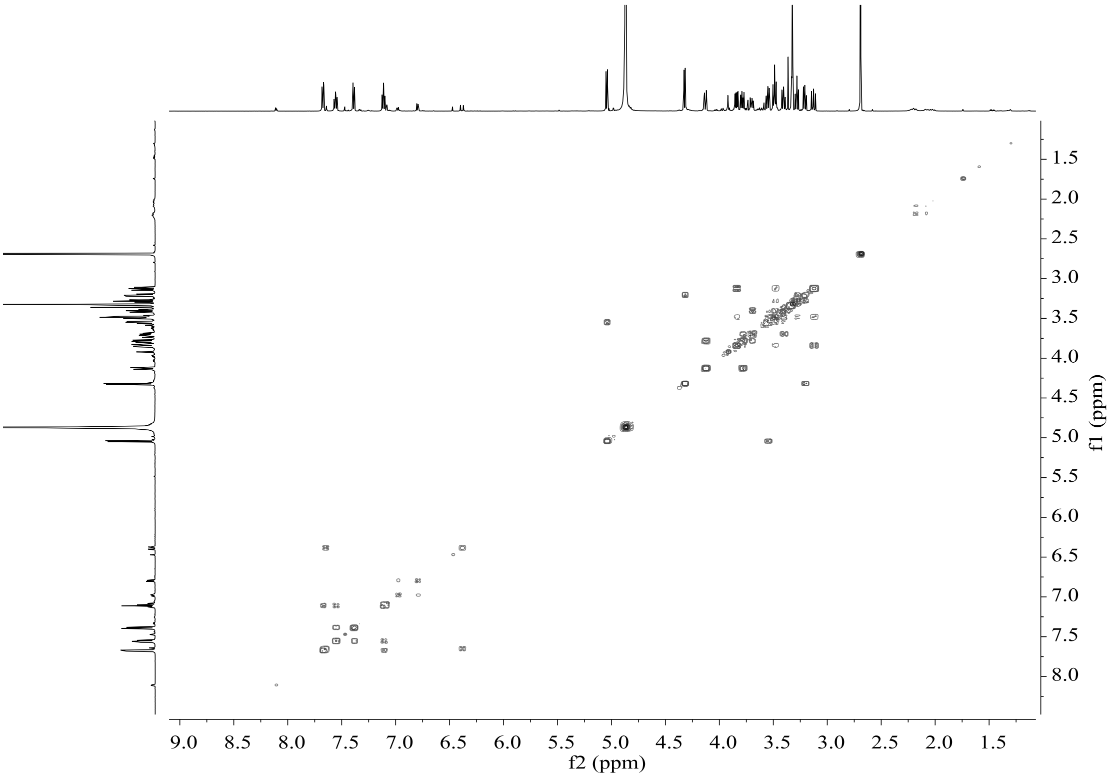

Figure S8. HMBC spectrum of **5** in CD_3OD

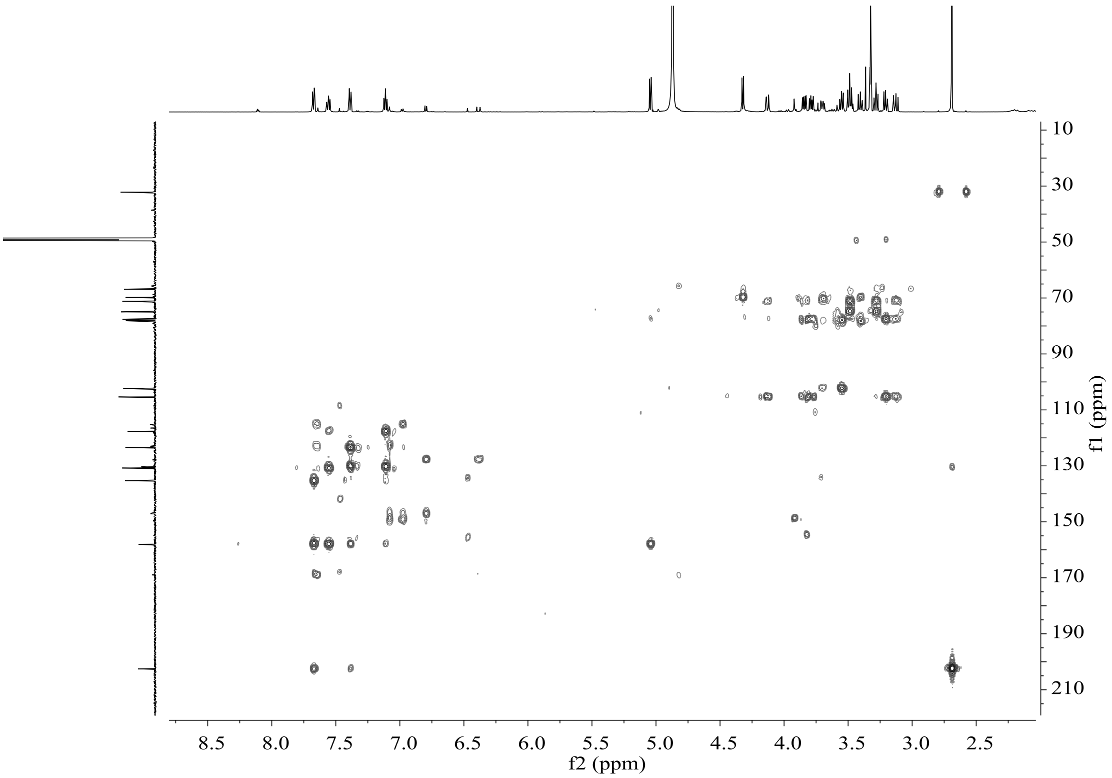

Figure S9. NOESY spectrum of **5** in CD_3OD

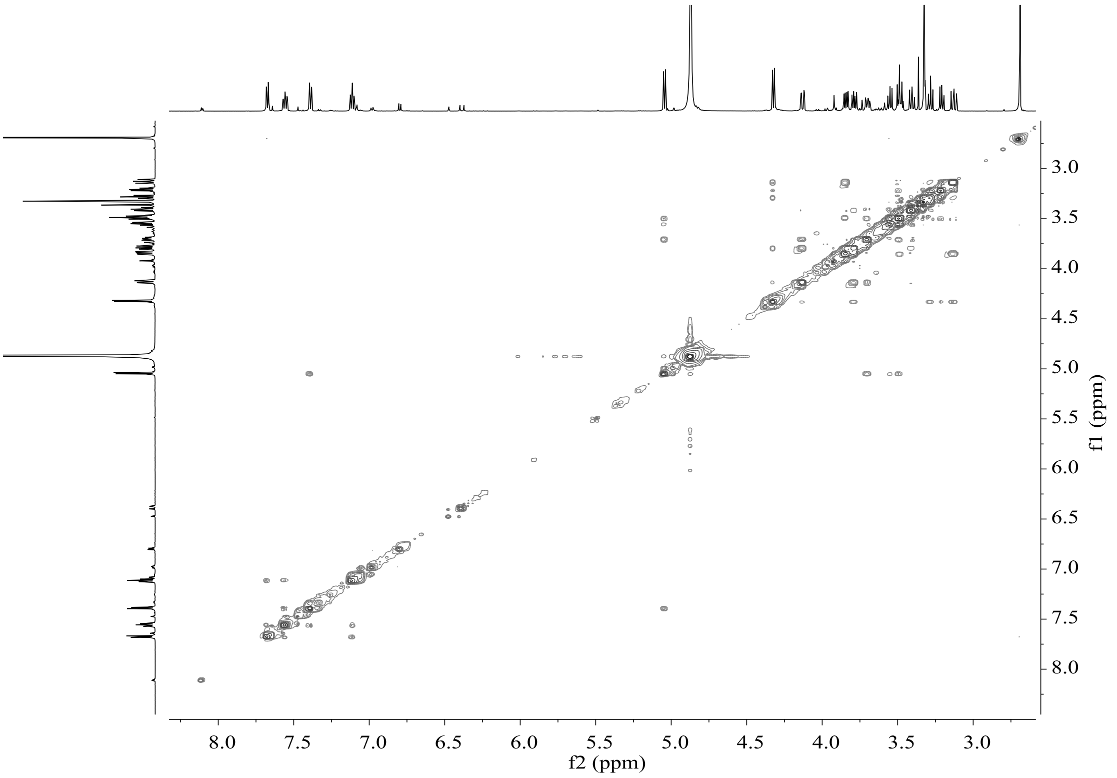

Supplement: Supplementary file 1 [file antioxidants-10-00652-s001.zip › antioxidants-1184633-supplementary.pdf]
